# Regional differences in the prevalence of generalized pustular psoriasis in Japan

**DOI:** 10.1111/1346-8138.17089

**Published:** 2024-01-31

**Authors:** Hideki Fujita, Ryoko Iwasaki, Satoshi Tsuboi, Yoko Murashiuma, Masashi Akiyama

**Affiliations:** ^1^ Department of Dermatology Nihon University School of Medicine Tokyo Japan; ^2^ Nippon Boehringer Ingelheim Co., Ltd Tokyo Japan; ^3^ Department of Dermatology Nagoya University Graduate School of Medicine Nagoya Japan

**Keywords:** epidemiology, Japan, prevalence, psoriasis, rare diseases

## Abstract

Generalized pustular psoriasis (GPP), a rare form of psoriasis, is characterized by neutrophil‐rich, sterile pustules. In Japan, GPP has intractable and rare disease designation, which allows patients to access support from national and local governments for medical expenses. Previously, similar numbers of patients in Tokyo and Hokkaido have been shown to have GPP designation, despite different population sizes. Here, we determine whether there are regional differences in the proportion of patients receiving GPP designation status in Japan and aim to identify causal factors. In this descriptive, retrospective cohort study, publicly available data were collected on the number of patients with intractable and rare disease designation for GPP in each prefecture and age classification (April 2018–March 2021). Three other designated intractable and rare disease cohorts were included: pemphigus, rare skin diseases, and all diseases. The primary outcome was the standardized morbidity ratio (SMR) of patients at prefecture level (observed numbers divided by expected). Regional differences were compared with the statistical expectation for the total population and age distribution of each prefecture. Regional differences were observed in all cohorts. Overall, 1910 patients had GPP as a designated intractable and rare disease in 2020. Regional differences in SMRs for GPP were observed with high SMRs (≥1.5) in Hokkaido, Tottori, Kagawa, and Miyazaki, and low SMRs (<0.6) in Gunma and Kanagawa. Regional differences in SMRs for GPP did not correlate with the number of medical doctors or dermatologists or internal migration. The number of medical doctors or dermatologists correlated with SMRs in the rare skin diseases and total cohorts. Regional differences in Japan exist in the number of patients with GPP who have an intractable and rare disease designation. Managing rare diseases is an important public health issue, and further research is required to elucidate the factors contributing to these differences.

## 1 INTRODUCTION

Generalized pustular psoriasis (GPP) is a rare form of psoriasis characterized by recurrent episodes of widespread eruption of sterile pustules.^1^, ^2^, ^3^ Repeated acute flares are painful and can be disfiguring and, due to the systemic nature of the disorder, the cutaneous symptoms are often accompanied by inflammatory symptoms, including fever, fatigue, and leukocytosis.^3^, ^4^, ^5^, ^6^ The clinical course of GPP is heterogeneous, with patients experiencing relapsing disease with recurrent flares or persistent disease with intermittent flares.^2^, ^6^ If left untreated, GPP may become life‐threatening.^7^

Estimating the global prevalence of GPP is challenging because of the heterogeneity of the condition; however, a recent review of epidemiology estimated that between 1.76 and 124 patients per million of the general population are affected by GPP, depending on their geographic region.^7^ GPP prevalence in patients with psoriasis was between 0.6% and 2.4%.^7^ Approximately 2000 people in Japan were recorded as having GPP at the end of 2020.^8^

The features of rare diseases, such as GPP, pose a challenge for health care professionals, researchers, legislators, policymakers, and patients; therefore, rare diseases are an important public health issue.^9^ Owing to the rarity and severity of the disease, GPP has been designated as an intractable and rare disease (known as *nanbyo* in Japanese) under the Act on Medical Care and Social Supports for Patients with Intractable and Rare Diseases, which was enacted in 2014 in Japan.^10^, ^11^, ^12^ As of November 2021, GPP was one of the 338 diseases to be designated as *nanbyo*.^8^, ^13^ This designation encourages a stable support system to be established that includes subsidizing the medical expenses for patients with intractable and rare diseases, allowing patients to access national government support to continue treatment.^10^ Application forms for designations must be completed by designated medical doctors.^10^ Every year, approximately 80 patients with moderate to severe GPP are newly eligible for specified medical expenses based on designation.^11^ The medical expenses of a patient designated with an intractable and rare disease in Japan are shared between national and the local governments in the 47 prefectures in Japan.^9^, ^14^

In 2020, despite Tokyo having a population twice the size of Hokkaido, the number of patients with GPP designated as having an intractable and rare disease in Hokkaido and Tokyo were similar (160 and 183, respectively).^12^, ^15^ Therefore, in this study we sought to investigate whether there are regional differences in the proportion of patients with GPP designation among the 47 prefectures across Japan. We also investigated whether regional differences were observed in all intractable, and rare diseases, and in intractable and rare skin diseases of interest. In this study, we also assessed regional differences in designation of pemphigus, a nonhereditary, intractable, and rare skin disease managed by dermatologists, as relatively similar numbers of patients with GPP and pemphigus received designation status in 2020 (GPP, *n* = 2058; pemphigus, *n* = 3515).^8^

## 2 METHODS

### 2.1 Study design and patients

This was a descriptive, retrospective epidemiological cohort study using data published by the government across the 47 prefectures of Japan during a period of 3 fiscal years (2018–2020) with data collection from April 2018 to March 2021.^16^, ^17^, ^18^, ^19^ Aggregated data of the number of patients with intractable and rare disease designation in each prefecture and age classification data were collected in four cohorts: GPP cohort: all patients with a GPP intractable and rare disease designation; pemphigus cohort: all patients with a pemphigus intractable and rare disease designation; rare skin disease of interest cohort: all patients with an intractable and rare skin disease designation (diseases were selected as per the Ministry of Health, Labour and Welfare [MHLW]‐funded research by the Rare and Interactable Skin Disease Research Group^20^ [GPP, pemphigus, pemphigoid, epidermolysis bullosa, congenital ichthyosis, pseudoxanthoma elasticum, and oculocutaneous albinism]); and total cohort: all patients with any intractable and rare disease designation.

All patients with an intractable and rare disease designation in Japan within the study period were included in the study. Patients with an unknown age were excluded.

The study does not include any individual data and, therefore, is outside the scope of ethics guidance by the Ministry of Health, Labour, and Welfare.

### 2.2 Outcomes

The primary outcome of the study was the standardized morbidity ratio (SMR) of patients at prefecture level in Japan in the four cohorts. The secondary outcome was the age distribution of patients at prefecture level in Japan in the four cohorts. Exploratory outcomes included the number of patients in the four cohorts per 100 000 people, assessment of correlation between SMRs in the four cohorts, and the number of medical doctors or dermatologists per 100 000 people, all at the prefecture level, and the correlation between internal migration of each prefecture and SMRs from the four cohorts.

### 2.3 Data sources

All data used in this study are publicly available and were aggregated population‐level data with no individual data included. Resident registration data were taken from the Ministry of Internal Affairs and Communications' annual report of the Japanese population in Japan.^19^ The statistics of Japanese designated intractable and rare diseases are reported every fiscal year by the Japan Intractable Diseases Information Center (JIDIC).^16^ Statistics on the number of medical doctors, dentists, and pharmacists are reported every 2 years by the MHLW in Japan, and the 2020 data were used in this analysis; the MHLW collect these data via a survey that all physicians in Japan are required to complete under the Medical Practitioners Act.^17^ Internal migration data from the Statistics Bureau of Japan for 2020 are used in the analysis.^18^

### 2.4 Bias

There is a delay between the actual occurrence of the outcomes and data availability from the government sources; therefore, to minimize information bias due to selecting a specific year, data were combined across 3 fiscal years.

### 2.5 Data analysis

The number of patients among the four cohorts were stratified by prefecture and at the national level over the study period (fiscal years 2018–2020). The regional difference of patients with intractable disease designation was compared with the statistical expectation in each prefecture. Statistical expectation was calculated based on the total population and age distribution of each prefecture, based on the hypothesis that intractable disease onset was equal throughout Japan.

SMRs were calculated by dividing the actual observed number of patients by the expected number of patients of each cohort across each prefecture. SMRs (± 95% confidence interval [CI]) were calculated to describe geographic differences across the four cohorts. The differences of age category in each prefecture are one of the major confounding factors and, therefore, the expected numbers of patients with these diseases in each cohort (at the prefecture level) each year were calculated with the rates of the diseases (at the national level) multiplied by the population of each prefecture for each age category. The total expected numbers over the 3‐year period were calculated.

Correlations between SMRs and possible associated factors were analyzed by calculating Pearson correlation coefficient. The examined associated factors were the number of medical doctors, dermatologists, and migration from or to each prefecture. Excel for Microsoft 365 MSO (16.0.14326.20908) and R version 4.1.1 (R Project for Statistical Computing) were used for the statistical analysis.

## 3 RESULTS

### 3.1 Patient population

Overall, in 2020, 946 110 patients had designated intractable and rare diseases: 1910, 3091, and 8387 patients had GPP, pemphigus, or rare skin disease designations, respectively. During the study period, the number of patients with designated intractable and rare diseases increased in all cohorts, except in the pemphigus cohort (Table 1). The number of patients with a GPP designation between 2018 and 2020 increased by 6.8%, whereas there was a 7.7% decline in the pemphigus cohort. Overall, there was a 10.6% increase in the number of patients in the rare skin disease of interest cohort, and a 6.0% increase in the number of patients with any designated intractable and rare disease over the same period.

**TABLE 1:** Number of patients with designated intractable and rare diseases stratified by age in Japan.

Patients, *n*	Cohort											
	Total cohort			Rare skin diseases^a^			GPP			Pemphigus		
	2018	2019	2020	2018	2019	2020	2018	2019	2020	2018	2019	2020
Total	892 445	912 714	946 110	7584	7906	8387	1788	1828	1910	3347	3152	3091
Age range, years												
0–9	742	619	550	27	22	17	4	3	1	2	2	1
10–19	7054	6254	6000	53	47	57	11	11	15	5	3	4
20–29	44 229	45 969	48 404	159	153	154	58	50	47	35	31	33
30–39	74 602	74 091	75 192	349	339	330	149	148	156	109	100	80
40–49	123 609	125 093	128 331	951	952	995	356	360	382	446	420	416
50–59	128 048	133 328	141 546	1213	1284	1382	346	350	368	616	619	633
60–69	184 713	179 747	177 674	1846	1800	1800	415	417	422	914	824	768
70–74	103 528	111 185	120 117	918	1034	1136	157	193	211	454	425	425
≥75	225 920	236 428	248 296	2068	2275	2516	292	296	308	766	728	731

**^a^Generalized pustular psoriasis (:** GPP), pemphigus, pemphigoid, epidermolysis bullosa, congenital ichthyosis, pseudoxanthoma elasticum, and oculocutaneous albinism.

Overall, Tokyo had the most patients with designated intractable and rare diseases of interest in 2020 among the four cohorts (Table S1). The prefectures with the highest number of patients in each of the cohorts were Tokyo (*n* = 774), Osaka (*n* = 749), Hokkaido (*n* = 575), and Hyogo (*n* = 501) in the rare skin disease of interest cohort; Tokyo (*n* = 162), Hokkaido (*n* = 152), Osaka (*n* = 156), and Aichi (*n* = 112) in the GPP cohort; Tokyo (*n* = 263), Osaka (*n* = 248), Hokkaido (*n* = 215), and Kanagawa (*n* = 185) in the pemphigus cohort; and Tokyo (*n* = 95 818), Osaka (*n* = 76 186), Kanagawa (*n* = 58 813), and Hokkaido (*n* = 54 166) in the total cohort.

### 3.2 SMR of patients with GPP intractable and rare disease designation

**FIGURE 1:**
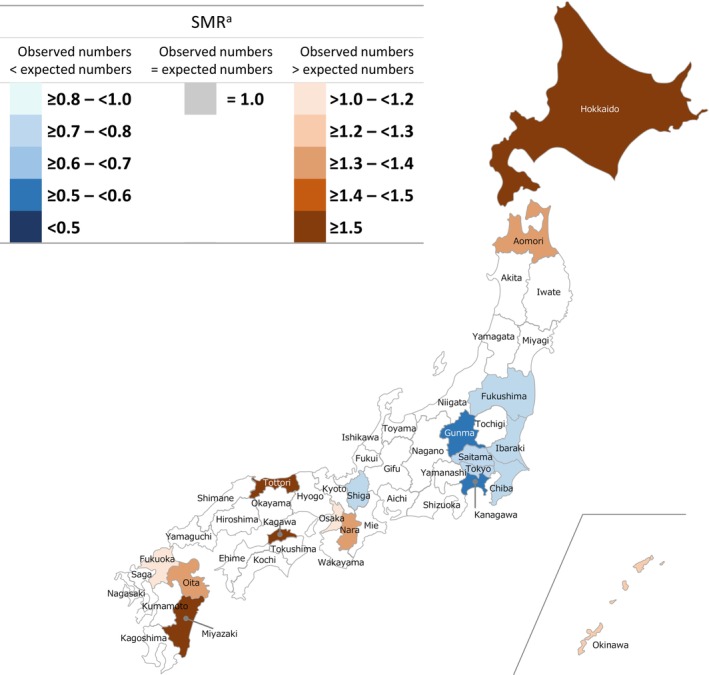
Standardized morbidity ratios (SMRs) for patients with generalized pustular psoriasis designated as an intractable and rare disease across all prefectures in Japan. ^a^Prefectures without a statistically significant difference in SMRs with the expected patient numbers are not colored on the map (29 of 47; 61.7%).

**FIGURE 2:**
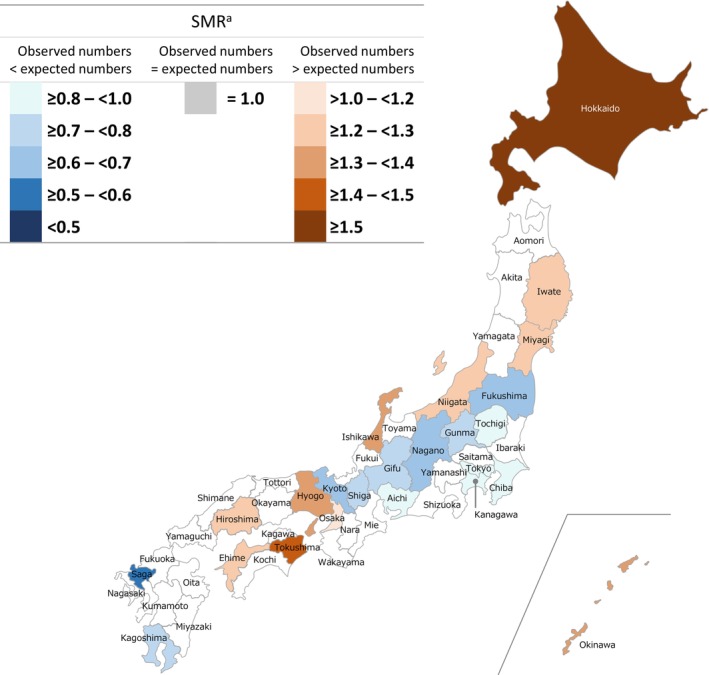
Standardized morbidity ratios (SMRs) for patients with pemphigus designated as an intractable and rare disease across all prefectures in Japan. ^a^Prefectures without a statistically significant difference in SMRs with the expected patient numbers are not colored on the map (23 of 47; 48.9%).

**FIGURE 3:**
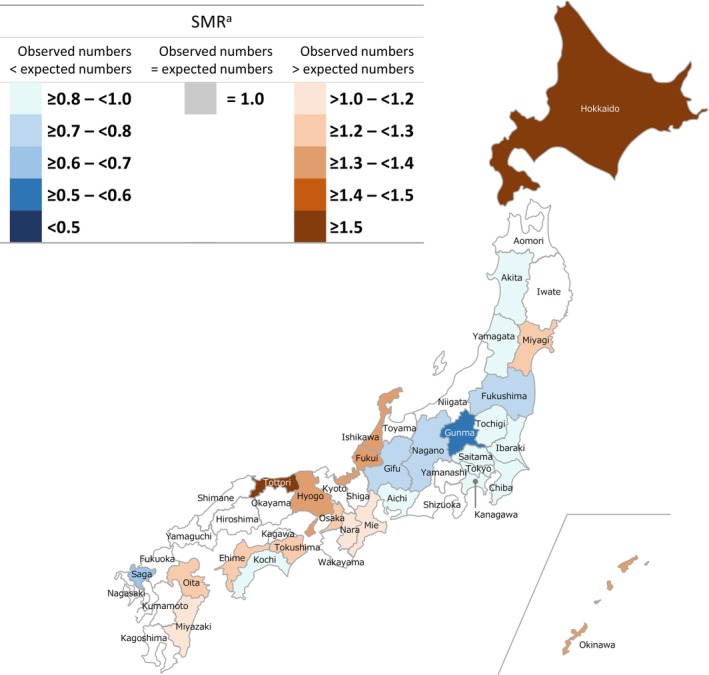
Standardized morbidity ratios (SMRs) for patients with intractable and rare skin^b^ diseases across all prefectures in Japan. ^a^Prefectures without a statistically significant difference in SMRs with the expected patient numbers are not colored on the map (18 of 47; 38.3%). ^b^Generalized pustular psoriasis, pemphigus, pemphigoid, epidermolysis bullosa, congenital ichthyosis, pseudoxanthoma elasticum, and oculocutaneous albinism.

**FIGURE 4:**
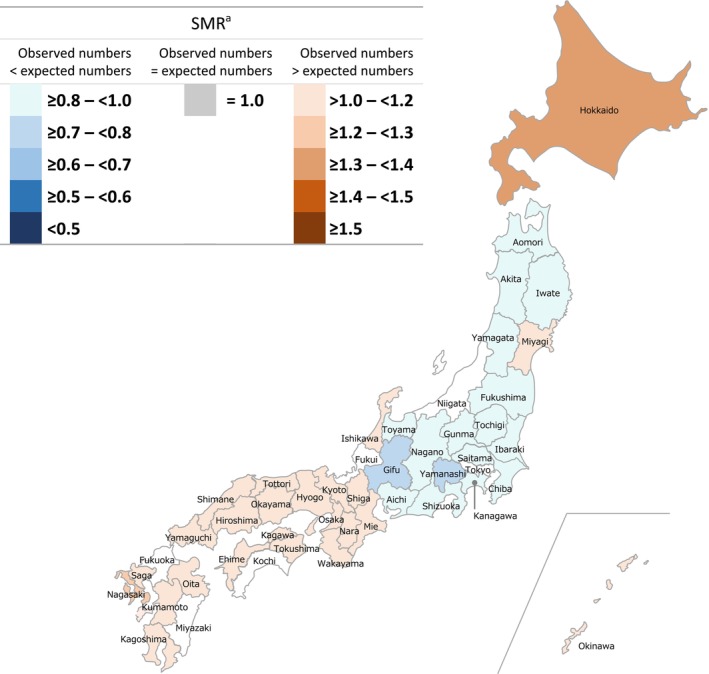
Standardized morbidity ratios (SMRs) for patients with any intractable and rare diseases across all prefectures in Japan. ^a^Prefectures without a statistically significant difference in SMRs with the expected patient numbers are not colored on the map (6 of 47; 12.8%).

Regional differences in SMRs for patients with intractable and rare diseases were observed among all cohorts. In the GPP cohort, no statistically significant differences between observed and expected morbidity rates were recorded in 29 of 47 (61.7%) prefectures (Figure 1); however, of the remaining prefectures, 10 had a high SMR and eight had a low SMR. Hokkaido, Tottori, Kagawa, and Miyazaki had the highest reported SMRs with ratios ≥1.5. Gunma and Kanagawa had the lowest reported SMRs, being ≥0.5 to <0.6 times lower than expected. In the pemphigus, rare skin diseases of interest, and total cohorts, respectively, 48.9%, 38.3%, and 12.8% of prefectures had no significant differences between observed and expected morbidity rates (Figures 2, 3, 4). Among all cohorts, there was a trend that SMRs from Western regions of Japan were higher than those observed in Eastern regions (except Hokkaido) (Figures 1, 2, 3, 4; Table S2).

### 3.3 Age distribution of patients with GPP intractable and rare disease designation

There was a trend toward increasing patient numbers with age among all cohorts (Table 1). The proportion of patients in the GPP cohort who were aged ≥70 years increased from 25% in 2018 to 27% in 2020.

During the study period, in the total cohort, there was a slight decline from 21% in 2018 to 19% in 2020 in the proportion of patients with intractable and rare diseases in the 60‐ to 69‐year age group. A similar decline was seen in the pemphigus cohort (from 27% to 25%) and rare skin diseases of interest cohort (from 24% to 21%), but not in the GPP cohort.

### 3.4 Number of patients with GPP intractable and rare disease designation per 100 000 people

The number of patients per 100 000 people with designated intractable and rare diseases, remained similar nationally between 2018 and 2020 after standardizing for the different prefecture populations (Table 2). Small increases were observed in all cohorts, except the pemphigus cohort, which declined slightly.

**TABLE 2:** Number of patients with designated intractable and rare diseases per 100 000 people at prefecture level in Japan.

Patients per 100 000 people, *n*	Cohort											
	Total cohort			Rare skin diseases^a^			GPP			Pemphigus		
	2018	2019	2020	2018	2019	2020	2018	2019	2020	2018	2019	2020
Total	712.8	731.5	761.3	6.1	6.3	6.7	1.4	1.5	1.5	2.7	2.5	2.5
Prefecture												
Hokkaido	974.8	1009.6	1036.5	9.8	10.6	11.0	2.6	2.8	2.9	4.7	4.5	4.1
Aomori	721.6	731.8	748.1	6.6	6.6	6.9	2.0	2.0	2.0	2.5	2.6	2.8
Iwate	730.7	734.9	754.0	6.0	6.6	8.1	1.7	1.7	1.9	3.5	3.4	3.6
Miyagi	773.1	767.8	797.2	7.8	7.4	7.4	1.5	1.7	1.7	3.8	3.1	2.9
Akita	783.6	739.7	766.5	8.9	4.5	4.9	1.6	1.3	1.4	6.0	1.9	1.1
Yamagata	621.0	653.0	670.0	5.1	6.2	6.4	1.0	1.1	1.2	3.0	3.1	3.0
Fukushima	670.5	700.0	716.7	4.4	5.1	4.5	1.2	1.2	1.2	1.8	1.9	1.6
Ibaraki	622.9	648.5	686.5	5.5	5.5	5.6	1.1	1.1	1.1	2.8	2.6	2.6
Tochigi	657.3	684.9	723.6	5.7	5.4	5.4	1.7	1.7	1.3	2.3	2.0	2.1
Gunma	662.4	685.2	705.5	4.3	4.0	4.1	0.9	0.9	0.9	2.3	1.7	1.7
Saitama	620.7	635.3	663.6	5.1	5.4	5.6	1.1	1.2	1.3	2.7	2.5	2.4
Chiba	681.5	676.1	702.4	5.5	5.5	5.6	1.2	1.1	1.1	2.3	2.3	2.1
Tokyo	689.9	701.8	722.7	5.1	5.5	5.8	1.1	1.1	1.2	2.2	2.2	2.0
Kanagawa	606.8	639.6	654.8	4.8	5.1	5.3	0.9	0.9	0.9	2.4	2.3	2.1
Niigata	752.4	805.8	760.9	6.5	7.8	6.4	1.8	1.9	1.7	3.2	3.8	2.8
Toyama	748.1	733.3	752.0	6.7	7.6	6.8	1.6	1.3	1.3	2.4	2.6	2.0
Ishikawa	788.2	785.4	817.5	8.5	8.6	8.7	1.8	1.9	2.0	3.8	3.4	2.8
Fukui	721.2	745.6	776.5	8.2	9.5	9.3	1.9	2.1	2.0	2.8	2.6	2.7
Yamanashi	524.9	561.4	564.8	5.1	6.5	5.4	1.1	1.5	1.5	2.5	2.4	2.3
Nagano	686.7	704.1	742.3	5.1	4.4	4.8	1.2	1.3	1.2	2.1	1.6	1.7
Gifu	556.5	575.4	597.1	4.8	5.0	4.9	1.4	1.5	1.6	2.1	2.1	1.8
Shizuoka	636.2	655.9	684.8	5.9	6.0	7.0	1.4	1.5	1.7	2.4	2.4	2.7
Aichi	562.3	568.0	592.5	4.9	5.0	5.5	1.4	1.3	1.5	1.9	2.1	2.0
Mie	763.9	773.6	815.1	6.4	6.9	8.8	1.5	1.6	1.9	2.4	2.4	3.0
Shiga	717.0	737.4	761.3	5.4	5.4	6.1	0.9	1.1	1.1	2.2	1.6	2.0
Kyoto	799.8	843.5	867.2	5.2	6.2	7.1	1.4	1.5	1.4	1.6	1.6	2.1
Osaka	765.8	815.4	886.2	6.8	7.4	8.7	1.6	1.7	1.8	2.9	2.6	2.9
Hyogo	705.9	744.5	794.6	7.7	8.7	9.2	1.4	1.5	1.8	3.3	3.3	3.3
Nara	845.1	877.0	908.4	7.6	7.9	8.0	2.0	1.9	2.1	2.0	2.3	2.2
Wakayama	820.8	843.4	877.7	5.7	6.1	6.7	1.3	1.4	1.4	2.8	2.6	2.7
Tottori	762.4	789.7	821.8	9.7	10.3	11.3	2.5	2.7	2.7	2.8	2.0	3.6
Shimane	850.7	883.1	914.7	7.9	6.6	7.3	1.6	1.5	1.8	3.2	2.7	2.5
Okayama	848.7	855.0	891.2	6.8	6.4	6.2	1.4	1.8	1.7	2.7	2.6	2.7
Hiroshima	734.8	729.8	775.4	5.9	5.7	6.2	1.6	1.5	1.8	3.4	3.0	2.9
Yamaguchi	849.1	876.3	900.0	5.8	7.3	7.9	1.5	1.4	1.6	3.2	3.1	3.0
Tokushima	832.2	840.1	888.9	8.2	8.5	8.7	1.9	1.9	2.0	4.4	3.9	3.7
Kagawa	857.0	882.2	918.4	7.0	7.2	7.7	2.4	2.6	2.3	1.9	1.8	2.6
Ehime	845.1	773.4	814.6	9.0	7.5	8.9	1.4	1.1	1.0	4.3	3.0	3.2
Kochi	763.9	787.9	801.0	6.2	6.2	5.1	1.2	1.4	1.1	2.8	2.4	1.8
Fukuoka	703.2	710.2	748.4	5.5	5.7	6.6	1.6	1.7	1.7	2.2	2.2	2.5
Saga	754.6	769.7	799.4	4.0	4.3	5.0	1.6	1.6	1.6	1.1	1.6	1.8
Nagasaki	899.3	915.3	946.0	6.1	6.9	6.3	1.9	1.5	1.3	2.1	2.7	2.3
Kumamoto	791.7	823.1	852.9	5.2	6.2	7.2	1.4	1.5	1.5	2.3	2.7	2.8
Oita	834.6	880.8	936.5	7.7	8.6	8.8	1.6	2.1	2.2	3.5	3.1	3.1
Miyazaki	751.1	758.4	790.4	7.6	7.4	7.9	2.5	2.3	2.1	2.7	2.5	2.5
Kagoshima	807.0	816.2	849.4	5.3	5.9	6.7	1.3	1.5	1.7	2.1	1.9	2.0
Okinawa	699.4	708.4	733.1	8.0	7.1	8.1	1.9	1.7	1.6	3.2	2.7	2.9

**^a^Generalized pustular psoriasis (:** GPP), pemphigus, pemphigoid, epidermolysis bullosa, congenital ichthyosis, pseudoxanthoma elasticum, and oculocutaneous albinism.

### 3.5 Association between SMRs of GPP intractable and rare disease designation at prefecture level and the number of medical doctors or dermatologists

Regional differences in SMRs in the GPP and pemphigus cohorts did not correlate with the number of medical doctors or dermatologists per 100 000 people (Table S3). SMR regional differences in the rare skin diseases of interest and total cohorts correlated with the number of medical doctors per 100 000 people (0.30; 95% CI, 0.02, 0.54 [*p* < 0.05] and 0.61; 95% CI, 0.39, 0.76 [*p* < 0.01], respectively) and the number of dermatologists per 100 000 people (0.29; 95% CI, 0.004, 0.53 [*p* < 0.05] and 0.32; 95% CI, 0.03, 0.55 [*p* < 0.05], respectively).

### 3.6 Association between SMRs of GPP intractable and rare disease designation at prefecture level and internal migration

There were no statistically significant correlations between the internal migration to or from other prefectures and SMRs of GPP, rare skin diseases of interest, and total designated intractable and rare diseases cohorts (Table S4). In the pemphigus cohort, SMRs correlated with migration to (−0.38 [95% CI, −0.60, 0.11] *p* < 0.01) but not from (−0.28 [95% CI, −0.52, 0.01]) other prefectures.

## 4 DISCUSSION

Regional differences in SMRs in patients with GPP designation were demonstrated in this analysis. SMRs >1.5 in some prefectures indicated that the patient numbers in these regions were 1.5 times higher than expected. The study provided an opportunity to assess whether the regional differences observed for GPP were also reflected in local variations for other designated intractable and rare diseases in Japan. Regional differences were also observed in the other cohorts, although the range of SMRs in the total cohort was narrower than that for the GPP cohort (total cohort, 0.7–1.3; GPP cohort, 0.6–1.8). Variation was also seen in the pemphigus cohort, which was included because of the similarities between these conditions.

Although the coronavirus disease 2019 (COVID‐19) pandemic has previously been shown to affect patient behavior in accessing health care,^21^ we did not observe a decrease in the number of patients receiving intractable disease designation from 2019 to 2020. In fact, we observed an increase of 5.5% in GPP designation from 2019 to 2020. A single‐center study by Uchida et al also demonstrated an increase in patients newly diagnosed with GPP from 2019 to 2020.^22^ The limited effect of the COVID‐19 pandemic on GPP diagnoses likely reflected the life‐threatening and systemic nature of GPP in which patients have limited choices other than seeking medical attention.

Several factors may contribute to the differences in SMRs observed. Prior to the adoption of the system for the designation of intractable diseases, support for patients with intractable diseases was provided independently by some prefectures.^23^ The extent of measures previously in place to manage intractable diseases may have contributed to our study results. For example, it may explain why although in general, SMRs were higher in Western Japan than in Eastern Japan, Hokkaido had consistently high SMRs in all cohorts. Hokkaido has a history of supporting patients with intractable diseases with the early establishment of the Hokkaido Intractable Disease Consultation and Support Center, which subsequently became a model for other centers across Japan.^24^ As a result, Hokkaido is better placed to manage patients with intractable and rare diseases including GPP effectively than other prefectures.

Access to medical resources may also partly explain the regional differences in SMRs observed in our study. As the number of designated hospitals in each prefecture for intractable and rare disease designation is limited, access to that hospital by patients may cause regional differences, especially in rural areas where public transport may be limited. Having access to large, specialized hospitals providing specialist medical services and diagnosis may also contribute to patient outcomes and, thus, the regional differences seen in SMRs. However, this hypothesis cannot explain why Tokyo has a lower SMR than Hokkaido, when Tokyo has the highest number of large and advanced hospitals in Japan. The differences in SMRs seen between Western and Eastern Japan may also be attributed to a higher number of hospitals, beds, and health care professionals in Western Japan than in Eastern Japan.^25^, ^26^

The number of medical doctors or dermatologists per 100 000 people of each prefecture was shown to correlate with SMRs in the total and rare skin disease of interest cohorts but not in the GPP and pemphigus cohorts. The small cohort sizes could account for the failure to associate health care professional numbers and SMRs for GPP and pemphigus. However, it may also be that the rarity of these diseases means that the general dermatologists included in our analysis have limited experience diagnosing these conditions, and patients may have been incorrectly diagnosed in prefectures with low SMRs. It would be valuable to assess in the future whether the number of physicians with expertise in psoriasis was associated with the regional differences seen in this study.

Our analysis was not able to identify the reasons for the differences between prefectures for the GPP cohort. However, we can speculate that causal factors specific to GPP, as well as those related to the intractable and rare skin diseases designation system described earlier, may explain the findings. *IL36RN* gene mutations have been shown to play a pathogenic role in patients with GPP,^27^, ^28^ and other genes including *CARD14*, *SERPINA3*, *AP1S3*, and *MPO* have been implicated in the development of GPP.^29^ It is plausible that the prevalence of familial mutations might not be uniform throughout Japan and may influence the regional differences observed. Of note, Hokkaido, Kochi, and Okinawa are known to have less migration than other regions, and therefore may be more susceptible to hereditary mutations. Although no correlation between migration and SMRs in the GPP cohort was shown in this study, this may be a result of the small numbers included. A further explanation could be that some patients with GPP could have had a history of psoriasis vulgaris, a relatively common skin condition.^5^, ^30^ The mechanism behind the occurrence of GPP in patients with psoriasis vulgaris is unknown; however, it is possible that if the medications more often used to treat psoriasis vulgaris in some prefectures were also beneficial in GPP, then this could explain the observed differences in SMRs.

This study provided some useful insights into the characteristics of patients with GPP. Although the number of patients with GPP designation increased during the 3‐year study period, the proportion of patients per 100 000 people in each prefecture showed only slight variation. There was a general trend among all cohorts increasing with age; however, this was interrupted in the group aged 70 to 74 years, which may reflect the lower birth rate in the early 1950s, following the increase in births after World War II.^31^ In Japan, other government funding systems are available for pediatric patients with chronic diseases, including GPP. However, if pediatric patients are designated in another government funding system, they will not apply for the JIDIC intractable disease designation, and, as a result, some pediatric patients will not have been captured in our analysis. For example, based on the data provided by the Information Center for Specific Pediatric Chronic Diseases in fiscal year 2018, 396 patients with chronic skin diseases applied for this pediatric chronic disease designation, although the number with specific skin diseases such as GPP are not disclosed.^32^

Our analysis had some limitations. Only patients diagnosed with moderate or severe GPP can apply for the intractable and rare disease designation; those with mild GPP cannot apply and, therefore, the number of GPP designations is not equal to the number of patients with GPP. To determine the regional epidemiology solely for the onset of GPP, data based on GPP diagnosis, not GPP designation, would need to be collected and analyzed. GPP diagnosis criteria in Japan^5^ are different from those in other countries, for example in Europe,^3^ and, as this study is a local study in Japan, the findings cannot be generalized to other countries. However, the methodology used could be generalized to regional epidemiology studies of GPP outside of Japan and to study other intractable and rare diseases. In addition to those discussed above, another limitation of our analysis is that for the number of dermatologists in Japan, it cannot be confirmed whether dermatologists captured in the data set specialized in cosmetic dermatology only and were therefore unequipped to treat the rare skin diseases of interest.

In conclusion, regional differences in Japan exist in the SMRs of patients with an intractable and rare disease designation for GPP. This difference did not correlate with the number of medical doctors or dermatologists across regions, and further research is warranted to elucidate the factors contributing to the variation. Rare diseases, such as GPP, are an important public health issue, and understanding the designation status and the potential factors that may influence this will be valuable.

## FUNDING INFORMATION

The study was supported and funded by Boehringer Ingelheim.

## CONFLICT OF INTEREST STATEMENT

Hideki Fujita declares receiving honoraria or fees for serving on advisory boards, as a speaker, and as a consultant, as well as grants as an investigator from AbbVie, Amgen, Boehringer Ingelheim, Bristol Myers Squibb, Daiichi Sankyo, Eisai, Eli Lilly, Janssen Pharmaceuticals, Japan Blood Products Organization, JMEC, Kaken Pharmaceutical, Kyowa Kirin, LEO Pharma, Mauro, Mitsubishi Tanabe Pharma, Nihon Pharmaceutical, Novartis, Otsuka Pharmaceutical, Sanofi, Sato Pharmaceutical, Sun Pharmaceutical Industries, Taiho Pharmaceutical, Torii Pharmaceutical, Towa Pharmaceutical, UCB, and Ushio, and is an Editorial Board member of The Journal of Dermatology and a co‐author of this article. To minimize bias, he was excluded from all editorial decision‐making related to the acceptance of this article for publication. Masashi Akiyama declares receiving lecture fees from Sanofi KK and Mauro, clinical research funding from Novartis Pharma KK and Boehringer Ingelheim, grant donations from AbbVie GK, Mauro, Ono Pharmaceutical, Sun Pharma Japan, Tanabe Mitsubishi, and Taiho Pharmaceutical, and is an Editorial Board member of The Journal of Dermatology and a co‐author of this article. To minimize bias, he was excluded from all editorial decision‐making related to the acceptance of this article for publication. Ryoko Iwasaki, Satoshi Tsuboi, and Yoko Murashiuma are employees of Boehringer Ingelheim.

## Supporting information


Tables S1‐S4

## Data Availability

We conducted a retrospective cohort study using publicly available data from the MHLW and Statistics Bureau of Japan. This study did not use individual patient data; therefore, informed consent was not necessary.
