# Supplementary material for: Regional differences in the prevalence of generalized pustular psoriasis in Japan
Source: J Dermatol. 2024 Jan 31;51(3):380–90. doi: 10.1111/1346-8138.17089 (PMC11483897; doi:10.1111/1346-8138.17089)
Supplement: Supplementary file 1 — Tables S1‐S4 [file JDE-51--s001.docx]

**Regional differences in the prevalence of generalized pustular psoriasis in Japan**

Hideki Fujita^1^ │ Ryoko Iwasaki^2^ │ Satoshi Tsuboi^2^ │ Yoko Murashiuma^2^ │ Masashi Akiyama^3^

^1^Department of Dermatology, Nihon University School of Medicine, Tokyo, Japan ^2^Nippon Boehringer Ingelheim Co., Ltd, Tokyo, Japan
^3^Department of Dermatology, Nagoya University Graduate School of Medicine, Nagoya, y

**SUPPORTING INFORMATION**

**CONTENTS**

## SUPPORTING INFORMATION TABLE S1 Number of patients with designated intractable and rare diseases of interest in 2020 2

## SUPPORTING INFORMATION TABLE S2 SMRs of patients with intractable and rare diseases at prefecture level in Japan 6

## SUPPORTING INFORMATION TABLE S3 SMRs correlation of designated intractable and rare diseases with the number of medical doctors or dermatologists per 100 000 people at prefecture level in Japan 9

## SUPPORTING INFORMATION TABLE S4 SMRs correlation of designated intractable and rare diseases with internal migration at prefecture level in Japan 10

**SUPPORTING INFORMATION**

**SUPPORTING INFORMATION TABLE S1** Number of patients with designated intractable and rare diseases of interest in 2020

| **Patients, *n*** | **Disease name** | | | | | | | | |
| --- | --- | --- | --- | --- | --- | --- | --- | --- | --- |
|  | **Total** | **Rare skin diseases of interest** | **GPP** | **Pemphigus** | **Epidermolysis bullosa** | **Congenital ichthyosis** | **Pemphigoid** | **OCA** | **PXE** |
| Total | 946 110 | 8387 | 1910 | 3091 | 302 | 76 | 2915 | 20 | 73 |
| Prefecture |  |  |  |  |  |  |  |  |  |
| Hokkaido | 54 166 | 575 | 152 | 215 | 18 | 10 | 178 | 1 | 1 |
| Aomori | 9497 | 87 | 26 | 35 | 3 | – | 23 | – | – |
| Iwate | 9255 | 99 | 23 | 44 | 1 | – | 30 | 1 | – |
| Miyagi | 18 087 | 168 | 39 | 66 | 3 | – | 59 | – | 1 |
| Akita | 7520 | 48 | 14 | 11 | 1 | 1 | 20 | – | 1 |
| Yamagata | 7198 | 69 | 13 | 32 | 1 | – | 23 | – | – |
| Fukushima | 13 377 | 84 | 22 | 30 | 6 | 1 | 24 | – | 1 |
| Ibaraki | 19 577 | 159 | 31 | 73 | 7 | 3 | 44 | 1 | – |
| Tochigi | 13 912 | 103 | 25 | 40 | 5 | – | 33 | – | – |
| Gunma | 13 470 | 78 | 18 | 33 | 7 | – | 20 | – | – |
| Saitama | 47 763 | 404 | 90 | 176 | 6 | 4 | 124 | – | 4 |
| Chiba | 43 230 | 346 | 70 | 130 | 16 | 4 | 115 | 3 | 8 |
| Tokyo | 95 818 | 774 | 162 | 263 | 29 | 4 | 306 | 4 | 6 |
| Kanagawa | 58 813 | 480 | 85 | 185 | 16 | 6 | 188 | – | – |
| Niigata | 16 873 | 141 | 37 | 62 | 1 | 1 | 40 | – | – |
| Toyama | 7795 | 70 | 13 | 21 | 5 | 2 | 27 | – | 2 |
| Ishikawa | 9181 | 98 | 22 | 32 | 4 | 1 | 36 | – | 3 |
| Fukui | 5939 | 71 | 15 | 21 | 3 | 1 | 31 | – | – |
| Yamanashi | 4574 | 44 | 12 | 19 | – | – | 13 | – | – |
| Nagano | 15 216 | 98 | 25 | 35 | 6 | – | 30 | – | 2 |
| Gifu | 11 786 | 97 | 31 | 36 | 4 | – | 26 | – | – |
| Shizuoka | 24 732 | 254 | 63 | 96 | 9 | 2 | 84 | – | – |
| Aichi | 43 261 | 403 | 112 | 148 | 15 | 4 | 121 | – | 3 |
| Mie | 14 335 | 155 | 34 | 52 | 5 | 1 | 62 | 1 | – |
| Shiga | 10 566 | 85 | 15 | 28 | – | 1 | 41 | – | – |
| Kyoto | 21 523 | 175 | 34 | 52 | 13 | – | 72 | – | 4 |
| Osaka | 76 186 | 749 | 156 | 248 | 33 | 2 | 299 | 4 | 7 |
| Hyogo | 43 188 | 501 | 97 | 182 | 17 | 4 | 195 | 2 | 4 |
| Nara | 12 174 | 107 | 28 | 29 | 4 | 3 | 40 | 1 | 2 |
| Wakayama | 8313 | 63 | 13 | 26 | 1 | 1 | 19 | – | 3 |
| Tottori | 4571 | 63 | 15 | 20 | 4 | – | 22 | – | 2 |
| Shimane | 6133 | 49 | 12 | 17 | 1 | 2 | 17 | – | – |
| Okayama | 16 687 | 116 | 31 | 50 | 4 | – | 31 | – | – |
| Hiroshima | 21 484 | 173 | 49 | 79 | 4 | 3 | 34 | – | 4 |
| Yamaguchi | 12 170 | 107 | 22 | 40 | 1 | – | 42 | 1 | 1 |
| Tokushima | 6542 | 64 | 15 | 27 | 1 | 1 | 20 | – | – |
| Kagawa | 8883 | 74 | 22 | 25 | 5 | 2 | 19 | – | 1 |
| Ehime | 11 044 | 121 | 14 | 43 | 9 | 1 | 53 | – | 1 |
| Kochi | 5642 | 36 | 8 | 13 | 3 | 2 | 10 | – | – |
| Fukuoka | 37 772 | 334 | 88 | 125 | 9 | 2 | 104 | – | 6 |
| Saga | 6528 | 41 | 13 | 15 | 1 | – | 11 | – | 1 |
| Nagasaki | 12 676 | 85 | 18 | 31 | 8 | 3 | 23 | – | 2 |
| Kumamoto | 14 945 | 126 | 27 | 49 | 5 | 1 | 43 | – | 1 |
| Oita | 10 651 | 100 | 25 | 35 | 1 | – | 39 | – | – |
| Miyazaki | 8601 | 86 | 23 | 27 | 3 | 1 | 31 | – | 1 |
| Kagoshima | 13 745 | 108 | 27 | 32 | 2 | 1 | 45 | – | 1 |
| Okinawa | 10 711 | 119 | 24 | 43 | 2 | 1 | 48 | 1 | – |

Abbreviations: GPP, generalized pustular psoriasis; OCA, oculocutaneous albinism; PXE, pseudoxanthoma elasticum.

**SUPPORTING INFORMATION TABLE S2** SMRs of patients with intractable and rare diseases at prefecture level in Japan

| **SMR (95% CI)** | **Cohort** | | | |
| --- | --- | --- | --- | --- |
|  | **Total cohort** | **Rare skin diseases of interest^†^** | **GPP** | **Pemphigus** |
| Hokkaido | 1.31 (1.30, 1.31) | 1.55 (1.47, 1.62) | 1.80 (1.63, 1.98) | 1.63 (1.51, 1.75) |
| Aomori | 0.94 (0.93, 0.95) | 0.96 (0.85, 1.09) | 1.29 (1.03, 1.62) | 0.93 (0.76, 1.14) |
| Iwate | 0.95 (0.94, 0.96) | 1.00 (0.88, 1.13) | 1.15 (0.90, 1.47) | 1.26 (1.06, 1.50) |
| Miyagi | 1.07 (1.06, 1.07) | 1.19 (1.09, 1.29) | 1.12 (0.93, 1.35) | 1.28 (1.12, 1.46) |
| Akita | 0.93 (0.92, 0.94) | 0.83 (0.72, 0.96) | 0.90 (0.66, 1.22) | 1.04 (0.84, 1.28) |
| Yamagata | 0.83 (0.82, 0.84) | 0.85 (0.74, 0.98) | 0.72 (0.51, 1.01) | 1.10 (0.90, 1.34) |
| Fukushima | 0.92 (0.91, 0.93) | 0.70 (0.62, 0.79) | 0.77 (0.60, 0.99) | 0.65 (0.53, 0.80) |
| Ibaraki | 0.88 (0.87, 0.89) | 0.85 (0.78, 0.93) | 0.75 (0.61, 0.92) | 1.01 (0.88, 1.15) |
| Tochigi | 0.93 (0.92, 0.94) | 0.85 (0.76, 0.95) | 1.05 (0.85, 1.30) | 0.81 (0.68, 0.97) |
| Gunma | 0.92 (0.91, 0.92) | 0.63 (0.56, 0.72) | 0.61 (0.46, 0.81) | 0.73 (0.60, 0.88) |
| Saitama | 0.89 (0.88, 0.89) | 0.86 (0.81, 0.91) | 0.81 (0.71, 0.92) | 1.01 (0.93, 1.10) |
| Chiba | 0.94 (0.94, 0.95) | 0.88 (0.83, 0.93) | 0.76 (0.66, 0.87) | 0.88 (0.80, 0.97) |
| Tokyo | 1.00 (1.00, 1.01) | 0.93 (0.89, 0.97) | 0.80 (0.73, 0.88) | 0.89 (0.83, 0.95) |
| Kanagawa | 0.89 (0.88, 0.89) | 0.83 (0.78, 0.87) | 0.62 (0.55, 0.71) | 0.91 (0.84, 0.98) |
| Niigata | 1.00 (0.99, 1.01) | 1.01 (0.92, 1.11) | 1.17 (0.98, 1.41) | 1.20 (1.05, 1.38) |
| Toyama | 0.97 (0.96, 0.98) | 1.04 (0.91, 1.19) | 0.92 (0.68, 1.25) | 0.86 (0.68, 1.09) |
| Ishikawa | 1.08 (1.06, 1.09) | 1.33 (1.19, 1.50) | 1.28 (0.99, 1.64) | 1.30 (1.08, 1.57) |
| Fukui | 1.00 (0.99, 1.02) | 1.38 (1.20, 1.58) | 1.34 (1.00, 1.81) | 1.04 (0.81, 1.34) |
| Yamanashi | 0.73 (0.72, 0.74) | 0.85 (0.72, 1.01) | 0.90 (0.63, 1.27) | 0.92 (0.71, 1.19) |
| Nagano | 0.93 (0.93, 0.94) | 0.71 (0.63, 0.79) | 0.83 (0.66, 1.04) | 0.67 (0.55, 0.81) |
| Gifu | 0.77 (0.76, 0.78) | 0.75 (0.67, 0.84) | 1.00 (0.81, 1.24) | 0.77 (0.64, 0.93) |
| Shizuoka | 0.88 (0.87, 0.89) | 0.97 (0.90, 1.04) | 1.02 (0.88, 1.20) | 0.95 (0.84, 1.07) |
| Aichi | 0.82 (0.81, 0.82) | 0.85 (0.80, 0.90) | 0.98 (0.88, 1.10) | 0.83 (0.75, 0.91) |
| Mie | 1.05 (1.04, 1.06) | 1.13 (1.02, 1.25) | 1.14 (0.92, 1.41) | 0.99 (0.84, 1.18) |
| Shiga | 1.05 (1.04, 1.06) | 0.93 (0.82, 1.06) | 0.72 (0.53, 0.98) | 0.79 (0.63, 0.98) |
| Kyoto | 1.13 (1.12, 1.14) | 0.96 (0.87, 1.05) | 0.95 (0.78, 1.16) | 0.69 (0.58, 0.82) |
| Osaka | 1.14 (1.13, 1.14) | 1.22 (1.17, 1.28) | 1.16 (1.05, 1.27) | 1.12 (1.04, 1.20) |
| Hyogo | 1.02 (1.01, 1.03) | 1.34 (1.27, 1.42) | 1.07 (0.94, 1.21) | 1.30 (1.19, 1.41) |
| Nara | 1.16 (1.15, 1.17) | 1.18 (1.05, 1.32) | 1.32 (1.05, 1.65) | 0.81 (0.65, 1.00) |
| Wakayama | 1.10 (1.08, 1.11) | 0.90 (0.77, 1.04) | 0.89 (0.64, 1.23) | 0.99 (0.79, 1.25) |
| Tottori | 1.04 (1.03, 1.06) | 1.56 (1.34, 1.82) | 1.74 (1.28, 2.36) | 1.04 (0.78, 1.40) |
| Shimane | 1.13 (1.12, 1.15) | 1.06 (0.89, 1.24) | 1.08 (0.75, 1.53) | 1.02 (0.78, 1.34) |
| Okayama | 1.17 (1.16, 1.18) | 1.00 (0.90, 1.11) | 1.10 (0.89, 1.36) | 1.04 (0.88, 1.22) |
| Hiroshima | 1.01 (1.01, 1.02) | 0.93 (0.85, 1.01) | 1.11 (0.93, 1.32) | 1.20 (1.06, 1.36) |
| Yamaguchi | 1.12 (1.11, 1.13) | 1.01 (0.90, 1.14) | 0.99 (0.77, 1.28) | 1.11 (0.93, 1.33) |
| Tokushima | 1.10 (1.08, 1.12) | 1.23 (1.07, 1.43) | 1.26 (0.92, 1.71) | 1.45 (1.17, 1.79) |
| Kagawa | 1.18 (1.16, 1.19) | 1.10 (0.96, 1.26) | 1.63 (1.28, 2.06) | 0.80 (0.62, 1.04) |
| Ehime | 1.05 (1.04, 1.06) | 1.25 (1.12, 1.39) | 0.77 (0.57, 1.03) | 1.29 (1.09, 1.52) |
| Kochi | 0.99 (0.97, 1.00) | 0.83 (0.69, 0.99) | 0.82 (0.55, 1.20) | 0.83 (0.62, 1.11) |
| Fukuoka | 1.00 (1.00, 1.01) | 0.96 (0.90, 1.02) | 1.16 (1.02, 1.31) | 0.92 (0.83, 1.02) |
| Saga | 1.05 (1.04, 1.07) | 0.68 (0.56, 0.83) | 1.08 (0.78, 1.49) | 0.58 (0.41, 0.81) |
| Nagasaki | 1.20 (1.19, 1.21) | 0.95 (0.84, 1.08) | 1.04 (0.81, 1.34) | 0.88 (0.72, 1.08) |
| Kumamoto | 1.10 (1.09, 1.11) | 0.94 (0.84, 1.05) | 0.98 (0.78, 1.24) | 0.99 (0.83, 1.17) |
| Oita | 1.15 (1.14, 1.17) | 1.24 (1.10, 1.39) | 1.31 (1.02, 1.67) | 1.19 (0.99, 1.44) |
| Miyazaki | 1.01 (1.00, 1.02) | 1.14 (1.00, 1.29) | 1.54 (1.22, 1.94) | 0.95 (0.76, 1.18) |
| Kagoshima | 1.09 (1.08, 1.10) | 0.89 (0.79, 1.00) | 1.01 (0.80, 1.27) | 0.75 (0.61, 0.91) |
| Okinawa | 1.09 (1.08, 1.10) | 1.39 (1.25, 1.55) | 1.29 (1.02, 1.62) | 1.30 (1.09, 1.55) |

*Note*: Average SMR values calculated from fiscal years 2018 to 2020.

Abbreviations: CI, confidence interval; GPP, generalized pustular psoriasis; SMR, standardized morbidity ratio.

^†^GPP, pemphigus, pemphigoid, epidermolysis bullosa, congenital ichthyosis, pseudoxanthoma elasticum, and oculocutaneous albinism.

**SUPPORTING INFORMATION TABLE S3** SMRs correlation of designated intractable and rare diseases with the number of medical doctors or dermatologists per 100 000 people at prefecture level in Japan

| **SMR (95% CI)** | **Cohort** | | | |
| --- | --- | --- | --- | --- |
|  | **Total cohort** | **Rare skin diseases^†^** | **GPP** | **Pemphigus** |
| Medical doctors^‡^  (per 100 000 people) | 0.61** (0.39, 0.76) | 0.30* (0.02, 0.54) | 0.28 (–0.01, 0.52) | 0.04 (–0.25, 0.33) |
| Dermatologists^‡^  (per 100 000 people) | 0.32* (0.03, 0.55) | 0.29* (0.004, 0.53) | 0.21 (–0.09, 0.47) | 0.13 (–0.17, 0.40) |

*Note*: SMR values calculated from fiscal years 2018 to 2020. For the number of medical doctors, data from 2020 was used.

Abbreviations: CI, confidence interval; GPP, generalized pustular psoriasis; SMR, standardized morbidity ratio.
*p<0.05; **p<0.01; ^†^GPP, pemphigus, pemphigoid, epidermolysis bullosa, congenital ichthyosis, pseudoxanthoma elasticum, and oculocutaneous albinism; ^‡^Medical doctors who worked for a medical institute in 2020, as reported by the Ministry of Health, Labour, and Welfare in Japan.

**SUPPORTING INFORMATION TABLE S4** SMRs correlation of designated intractable and rare diseases with internal migration at prefecture level in Japan

| **SMR (95% CI)** | **Cohort** | | | |
| --- | --- | --- | --- | --- |
|  | **Total cohort** | **Rare skin diseases^†^** | **GPP** | **Pemphigus** |
| Migration from other prefectures^‡^ | −0.07 (−0.35, 0.23) | −0.16 (−0.43, 0.14) | −0.28 (−0.52, 0.01) | −0.28 (−0.52, 0.01) |
| Migration to other prefectures^‡^ | −0.04 (−0.32, 0.25) | −0.23 (−0.48, 0.06) | −0.27 (−0.52, 0.02) | −0.38* (−0.60, −0.11) |

*Note*: SMR values calculated from fiscal years 2018 to 2020. For the number of internal migrations, data from 2020 was used.

Abbreviations: CI, confidence interval; GPP, generalized pustular psoriasis; SMR, standardized morbidity ratio.
*p<0.01; ^†^GPP, pemphigus, pemphigoid, epidermolysis bullosa, congenital ichthyosis, pseudoxanthoma elasticum, and oculocutaneous albinism; ^‡^Internal migration data in 2020, as reported by the Statistics Bureau of Japan.
